# Lifestyle Factors and Breast Cancer in Females with PTEN Hamartoma Tumor Syndrome (PHTS)

**DOI:** 10.3390/cancers16050953

**Published:** 2024-02-27

**Authors:** Linda A. J. Hendricks, Katja C. J. Verbeek, Janneke H. M. Schuurs-Hoeijmakers, Arjen R. Mensenkamp, Hilde Brems, Robin de Putter, Violetta C. Anastasiadou, Marie-Charlotte Villy, Arne Jahn, Verena Steinke-Lange, Margherita Baldassarri, Arvids Irmejs, Mirjam M. de Jong, Thera P. Links, Edward M. Leter, Daniëlle G. M. Bosch, Hildegunn Høberg-Vetti, Marianne Tveit Haavind, Kjersti Jørgensen, Lovise Mæhle, Ana Blatnik, Joan Brunet, Esther Darder, Emma Tham, Nicoline Hoogerbrugge, Janet R. Vos

**Affiliations:** 1 Department of Human Genetics, Radboudumc Expert Center for PHTS, Radboud University Medical Center, 6525 GA Nijmegen, The Netherlands; 2 Radboud Institute for Medical Innovation, Radboud University Medical Center, 6525 GA Nijmegen, The Netherlands; 3 Department of Human Genetics, University of Leuven, 3000 Leuven, Belgium; hilde.brems@kuleuven.be; 4 Center for Medical Genetics, Ghent University Hospital, 9000 Ghent, Belgium; 5 Karaiskakio Foundation, Nicosia Cyprus and Archbishop Makarios III Children’s Hospital, Nicosia 2012, Cyprus; 6 Service de Génétique, Institut Curie, 75248 Paris, France; 7 Institute for Clinical Genetics, Faculty of Medicine Carl Gustav Carus, Technische Universitat Dresden, 01062 Dresden, Germany; 8 Hereditary Cancer Syndrome Center Dresden, 01307 Dresden, Germany; 9 German Cancer Consortium (DKTK), 69120 Dresden, Germany; 10 Medical Genetics Center, 80335 Munich, Germany; 11 Arbeitsgruppe Erbliche Gastrointestinale Tumore, Medizinische Klinik und Poliklinik IV—Campus Innenstadt, Klinikum der Universität München, 81377 Munich, Germany; 12 Medical Genetics, University of Siena, 53100 Siena, Italy; 13 Med Biotech Hub and Competence Center, Department of Medical Biotechnologies, University of Siena, 53100 Siena, Italy; 14 Genetica Medica, Azienda Ospedaliero-Universitaria Senese, 53100 Siena, Italy; 15 Institute of Oncology, Riga Stradins University, 1007 Riga, Latvia; 16 Breast Unit, Pauls Stradins Clinical University Hospital, 1002 Riga, Latvia; 17 Department of Genetics, University of Groningen, University Medical Center Groningen, 9713 GZ Groningen, The Netherlands; 18 Department of Endocrinology, University of Groningen, University Medical Center Groningen, 9713 GZ Groningen, The Netherlands; t.p.links@umcg.nl; 19 Department of Clinical Genetics, Maastricht University Medical Center, 6229 ER Maastricht, The Netherlands; 20 Department of Clinical Genetics, Erasmus MC Rotterdam, 3015 GD Rotterdam, The Netherlands; 21 Western Norway Familial Cancer Center, Department of Medical Genetics, Haukeland University Hospital, 5021 Bergen, Norway; 22 Department of Medical Genetics, Oslo University Hospital, 0450 Oslo, Norway; 23 Department of Clinical Cancer Genetics, Institute of Oncology Ljubljana, 1000 Ljubljana, Slovenia; 24 Hereditary Cancer Program, Catalan Institute of Oncology, IDIBELL-IDIBGI, 08916 Barcelona, Spain; 25 Department of Clinical Genetics, Karolinska University Hospital, 14186 Stockholm, Sweden; 26 Department of Molecular Medicine and Surgery, Karolinska Institute, 17177 Stockholm, Sweden

**Keywords:** alcohol drinking, body mass index, breast neoplasm, exercise, hamartoma syndrome, multiple

## Abstract

**Simple Summary:**

Females with PTEN Hamartoma Tumor Syndrome (PHTS) have very high hereditary breast cancer risks up to 76%. The aim of this European cohort study was to the describe the lifestyle in PHTS patients and to assess associations between physical activity, alcohol consumption, tobacco smoking, BMI and breast cancer in female adult PHTS patients. It was observed that of 125 patients who completed the questionnaire, 81% were ≥2 times/week physically active, 86% consumed on average <1 glass of alcohol/day, 78% never smoked and most patients were overweight or obese (72%). In total, 39 developed breast cancer (31%). No indications were found that associations between lifestyle and breast cancer in PHTS patients are different from the general population. These findings suggest that awareness about lifestyle among PHTS patients is important, as a healthier lifestyle could potentially decrease their breast cancer risk in a similar way as for the general population.

**Abstract:**

Females with PTEN Hamartoma Tumor Syndrome (PHTS) have breast cancer risks up to 76%. This study assessed associations between breast cancer and lifestyle in European female adult PHTS patients. Data were collected via patient questionnaires (July 2020–March 2023) and genetic diagnoses from medical files. Associations between lifestyle and breast cancer were calculated using logistic regression corrected for age. Index patients with breast cancer before PHTS diagnosis (breast cancer index) were excluded for ascertainment bias correction. In total, 125 patients were included who completed the questionnaire at a mean age of 44 years (SD = 13). This included 21 breast cancer indexes (17%) and 39 females who developed breast cancer at 43 years (SD = 9). Breast cancer patients performed about 1.1 times less often 0–1 times/week physical activity than ≥2 times (OR_total-adj_ = 0.9 (95%CI 0.3–2.6); consumed daily about 1.2–1.8 times more often ≥1 than 0–1 glasses of alcohol (OR_total-adj_ = 1.2 (95%CI 0.4–4.0); OR_non-breastcancer-index-adj_ = 1.8 (95%CI 0.4–6.9); were about 1.04–1.3 times more often smokers than non-smokers (OR_total-adj_ = 1.04 (95%CI 0.4–2.8); OR_non-breastcancer-index-adj_ = 1.3 (95%CI 0.4–4.2)); and overweight or obesity (72%) was about 1.02–1.3 times less common (OR_total-adj_ = 0.98 (95%CI 0.4–2.6); OR_non-breastcancer-index-adj_ = 0.8 (95%CI 0.3–2.7)). Similar associations between lifestyle and breast cancer are suggested for PHTS and the general population. Despite not being statistically significant, results are clinically relevant and suggest that awareness of the effects of lifestyle on patients’ breast cancer risk is important.

## 1. Introduction

Females with PTEN Hamartoma Tumor Syndrome (PHTS) have a high hereditary risk of developing breast cancer due to a pathogenic *PTEN* germline variant [1,2,3]. Cancer risk estimates range from 54% to 76% by age 60 years [3]. PHTS patients have different breast cancer risks depending on their *PTEN* germline variant: truncating variants were associated with two to three times higher risks than missense variants, and variants located in the phosphatase domain were additionally associated with two times higher risks than variants in domain C2 [3]. The association between non-genetic factors and breast cancer has not been evaluated in PHTS yet. If these non-genetic factors contribute significantly, this might give patients self-management tools to lower their breast cancer risk. Especially, lifestyle counseling for factors that patients can manage themselves such as physical activity, alcohol consumption, tobacco smoking and body mass index (BMI) could possibly relatively easily lower their risk.

In a general population, it has been shown that decreased physical activity increases breast cancer risk in females, whereas overweight and obesity are associated with up to two times increased postmenopausal breast cancer risks [4,5,6]. The impact of alcohol consumption and tobacco smoking remains under debate. A 1.1 to 1.3 times increased breast cancer risk for smoking was reported, and a slightly increased breast cancer risk for alcohol consumption was reported [7,8,9,10,11], while others reported breast cancer risk reduction for alcohol consumption of less than 10 g of ethanol per day [5].

For females with a hereditary predisposition for breast cancer caused by a pathogenic germline variant in *BRCA1/2*, it has been shown that lifestyle and hormonal risk factors could affect their breast cancer risk. Physical activity is reported to lower breast cancer risks, and obesity is associated with increased postmenopausal breast cancer risks [12,13,14]. However, no association between alcohol consumption and breast cancer risk was observed [15,16]. Although a modest association between smoking and increased breast cancer risks was reported [8,17,18], others could not confirm this [19].

In females with PHTS, the association between lifestyle factors and breast cancer has not yet been studied, and an overview of the lifestyle in PHTS patients is lacking, while this knowledge is important for breast cancer management [20]. Therefore, the aim of this study was to describe the lifestyle and to assess the association between physical activity, alcohol consumption, tobacco smoking and BMI and breast cancer in female adult PHTS patients. 

## 2. Methods

### 2.1. Study Design

European female adult PHTS patients were retrospectively recruited via genetic centers and PHTS expert centers across Europe and via active self-recruitment. Patients were invited to complete a single questionnaire (either online or via paper) between July 2020 and March 2023 containing 61 items on demographics, PHTS diagnosis, medical history and lifestyle. Details about the *PTEN* germline variant and cancer stage were obtained from genetic centers. This study was approved by the institutional ethics committees, and written informed consent was obtained from all participants. 

### 2.2. Clinical, Genetic and Lifestyle Risk Factor Definitions

Physical activity was collected as the frequency one performs ≥30 minutes of at least moderately intensive exercise (such as walking, exercising or vacuuming). This was classified as <1 time/week, 1 time/week, 2–3 times/week, 4–5 times week and >5 times/week. 

Alcohol consumption was collected as the number of glasses of alcoholic beverage that are consumed on average during weekdays (Monday to Thursday) and weekend days (Friday to Sunday). In addition, the average number of glasses per day was calculated. 

Tobacco smoking behavior was categorized as currently smoking, smoked in the past, never smoked, and never smoked but indoor smoking at home. The amount of cigarettes, cigars or tobacco pipes that patients were currently smoking or have smoked in the past per day were requested and categorized as usually <10, usually 10–25 and usually >25.

BMI was calculated based on requested information on height (in cm) and weight (in kg) and classified as underweight (BMI < 18.5 kg/m^2^), normal weight (BMI 18.5–24.9 kg/m^2^), overweight (BMI 25.0–29.9 kg/m^2^) and obesity (BMI ≥ 30.0 kg/m^2^).

The *PTEN* variant coding effect was categorized as truncating (including predicted truncating), missense or other, and protein domains according to spatial categorization (Supplementary Tables S1 and S2).

### 2.3. Statistical Analyses

Descriptive statistics were performed according to appropriate measures depending on data distribution. Associations between lifestyle factors and breast cancer were assessed using logistic regression with and without adjustment for age at questionnaire completion (i.e., last follow-up). The outcome consisted of the self-reported, first primary invasive or in situ breast cancer. 

An index patient is defined as the first identified patient in a family. The majority of index patients have symptoms related to PHTS at the time of their genetic test, because this was the reason they received genetic testing. A part of these index patients received genetic testing because they had breast cancer; these patients are referred to as breast cancer index patients (i.e., they had breast cancer before their PHTS diagnosis). Ascertainment bias correction was applied by excluding breast cancer index patients. OR-_total_ refers to uncorrected and OR_non-breastcancer-index_ to ascertainment bias corrected odds ratios (ORs). ORs corrected for age at questionnaire completion are presented as OR_total-adj_ and OR_non-breastcancer-index-adj._

Analyses were performed using RStudio (V.4.1.1). A two-sided *p*-value < 0.05 was considered statistically significant.

## 3. Results

### 3.1. Patient Population

Out of 146 female adult European PHTS patients who received a questionnaire invitation, 126 completed it (86%). Additionally, seven patients started the online questionnaire but did not complete the details regarding cancer history or lifestyle factors. One patient was excluded due to pregnancy, leaving 125 patients for analyses. 

Patients from 12 countries participated, of whom the majority of patients were Dutch (56%), Norwegian (19%) or Spanish (8%). Twenty-one (17%) patients were breast cancer index patients and were excluded in analyses corrected for ascertainment bias. The mean age at PHTS diagnosis and questionnaire completion was 37 years (SD = 14) and 44 years (SD = 13), respectively. In total, thirty-nine females (31%) developed breast cancer at a mean age of 43 years (SD = 9) (Table 1); this included 21 breast cancer index patients, of which 3 patients reported in situ breast cancer. The median time between first breast cancer diagnosis and questionnaire completion was 6 years (range 0–39). Stage was available for 36/39 diagnoses and 20% were in situ (stage 0), 42% stage I, 25% stage II, 11% stage III and 3% stage IV.

Detailed variant annotation was available for 124 patients (99%). Overall, 41% had a missense variant and 58% a truncating variant. Sixty-two percent had a variant located in the phosphatase domain and 26% in domain C2 (Table 1, Supplementary Tables S1 and S2). 

### 3.2. Physical Activity 

Most patients (39%) performed 2–3 times/week physical activity. This was similar when excluding breast cancer index patients (Table 1). 

**Figure 1:**
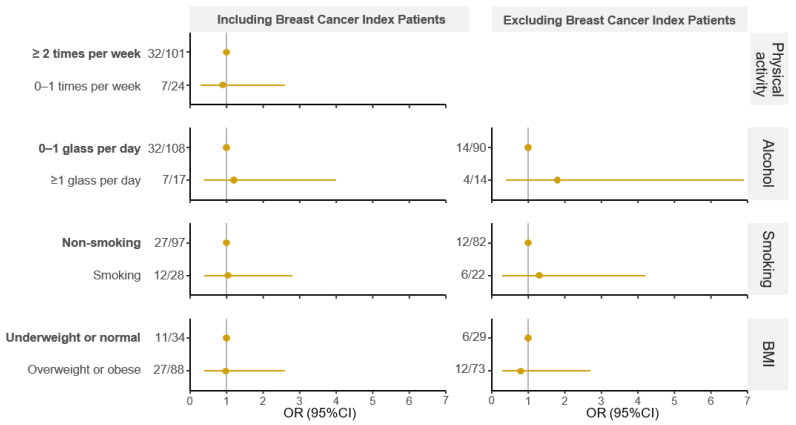
Odds ratios for the association between breast cancer and each lifestyle factor, adjusted for age at questionnaire completion. Odds ratios (ORs) are presented with corresponding 95% confidence intervals (95%CI). Non-smoking includes patients who have never smoked and those who have never smoked but had indoor smoking at home. Smoking includes patients who reported to be current smokers or smokers in the past. The reference category for each lifestyle factor is presented in bold. The vertical line indicates OR = 1.00, meaning no difference. For each group, the number of patients with breast cancer (n) and the total number of patients with the lifestyle risk factor (N) are presented (n/N).

Patients with breast cancer performed about 1.1 times less often 0–1 times per week physical activity than ≥2 times physical activity per week (OR_total-adj_ = 0.9, 95%CI 0.3–2.6) (Figure 1, Table 2). When excluding breast cancer index patients, the number of breast cancer events (n = 1) was too low for analysis. 

### 3.3. Alcohol Consumption

Overall, 80 (64%) patients reported total alcohol abstinence. Of the remaining patients, the majority (35/45, 78%) reported only alcohol consumption during weekend days. This was similar when excluding breast cancer index patients (Table 1). 

Patients with breast cancer had about 1.2 to 1.8 times more often alcohol consumption of ≥1 glass per day than 0–1 glass per day (OR_total-adj_ = 1.2, 95%CI 0.4–4.0; OR_non-breastcancer-index-adj_ = 1.8, 95%CI 0.4–6.9; Figure 1, Table 2).

### 3.4. Tobacco Smoking

Six (5%) patients currently smoke, 22 (18%) smoked in the past and 97 (78%) never smoked (including eight patients who never smoked but reported indoor smoking at home). The majority (68%) of the past and present smokers smoked less than 10 cigarettes, cigars or tobacco pipes per day. This was similar when excluding breast cancer index patients (Table 1). 

Patients with breast cancer were about 1.04 to 1.3 times more often smokers than non-smokers (OR_total-adj_ = 1.04, 95%CI 0.4–2.8; OR_non-breastcancer-index-adj_ = 1.3, 95%CI 0.3–4.2; Figure 1, Table 2).

### 3.5. Body Mass Index

Two (2%) patients were underweight, 32 (26%) had a normal weight, 38 (31%) were overweight, 50 (41%) were obese and BMI was missing for 3 patients. This was similar when excluding breast cancer index patients (Table 1). 

An indication of 1.02 to 1.3 times less often being overweight or obese than underweight or normal weight was observed for breast cancer patients (OR_total-adj_ = 0.98, 95%CI 0.4–2.6; OR_non-breastcancer-index-adj_ = 0.8, 95%CI 0.3–2.7; Figure 1, Table 2). 

### 3.6. Multiple Lifestyle Risk Factors

When considering physical activity, alcohol consumption, tobacco smoking and BMI, 5 (4%) patients reported no presumed risk-increasing lifestyle factors, 31 (25%) reported one, 68 (54%) reported two, 17 (14%) reported three and 4 (3%) reported four types of presumed risk-increasing lifestyle factors. 

## 4. Discussion

This is the first study that described the lifestyle and assessed the association between lifestyle factors (including physical activity, alcohol, tobacco smoking and BMI) and breast cancer in female PHTS patients. Our results emphasized that many among the female PHTS population have an unhealthy BMI: 73% were overweight, of which 41% were obese. Our results suggest that patients with breast cancer have a higher alcohol consumption and were more often smoking tobacco, although not statistically significant (ORs 1.2 to 1.8 and 1.04 to 1.3, respectively). No strong association was observed for physical activity and breast cancer diagnoses. 

Previous meta-analyses showed a 1.1 times decreased postmenopausal breast cancer risk in the general population for physical activity when comparing groups with highest to lowest activity levels [21]. Another meta-analysis indicated a small risk reduction per 7 metabolic equivalent task hours per week of 1.03 times (i.e., MET, normalized measure of physical activity) [21]. In our study, we compared 0–1 times of at least 30 minutes of moderate-intensive physical activity to ≥2 times exercise per week, and a slight reversed association with breast cancer was observed; however, ascertainment bias corrected analyses could not be performed. 

The majority of female adult PHTS patients reported overall alcohol abstinence (64%). However, self-reported alcohol consumption is known to be often underreported and maximally represents 75% of the real alcohol consumption [22]. Therefore, alcohol consumption by PHTS patients might be higher than we currently report. Previous meta-analysis showed that alcohol consumption possibly increases premenopausal breast cancer risks and increases postmenopausal breast cancer risks by 1.1 times per 10 g of ethanol per day (equivalent to 1 glass/day) [21]. This is a similar effect size of the suggested 1.2 to 1.8 times more often ≥1 glass of alcohol consumption per day for breast cancer patients in this study; however, directions of associations in this study remain uncertain due to the timing of questionnaire completion. 

Despite the fact that tobacco smoking is a known carcinogenic for, e.g., lung cancer, the association between smoking and breast cancer remains rather contradictory [23,24]. Various studies have reported a 1.1 times increased breast cancer risk for smokers [8]. This is in line with the observation that patients with breast cancer were suggested to be 1.04 to 1.3 times more often smokers versus non-smokers. 

Obesity is vastly more common in this female adult PHTS population (41%) than in the general European population (16%) [25]. A small statistically non-significant association of 1.02–1.3 times less often being overweight or obese than underweight or normal weight for breast cancer patients was observed. Despite that the direction of the association remains uncertain, this is a similar effect size, although reversed, to 1.1 to 2.0 times increased (postmenopausal) risks reported for the general population and *BRCA1/2* patients [6,14,26]. Despite the fact that we observed no statistically significant association with breast cancer and BMI, maintaining a normal weight is important for overall health [27,28]. Therefore, it is advised to motivate female PHTS patients to maintain their BMI in a healthy range. 

This is the first study to date that described the lifestyle and assessed the association between lifestyle factors and breast cancer in PHTS patients. Even though this was a substantial cohort for a rare disease, the number of cases and the study design remained limited; therefore, subcategories of lifestyle factors had to be aggregated, and causal effect and associations could not be distinguished. The relation between the various lifestyle factors and breast cancer risk are well described for the general population and other hereditary cancer predisposition syndromes. As indicated above, the observed associations in this study were in line with previously reported effect sizes for lifestyle factors and breast cancer in the general population and *BRCA1/2* patients. The majority of breast cancer diagnoses in this study were low stage (0–II, 87%). A previous study on changes in lifestyle behavior after ductal carcinoma in situ (stage 0) demonstrated a minimal increase of approximately two kilograms in body weight up to 6 years after diagnosis (in line with median time from breast cancer diagnosis to questionnaire completion of 6 years in the current study) [29]. Furthermore, no significant differences in alcohol consumption were observed after DCIS diagnosis, and a decrease of approximately 5% was observed in the percentage of patients smoking [29]. Another study emphasized that a progressive decrease in physical activity levels was observed with increasing breast cancer stage [30]. Considering the mainly low-stage breast cancers in this study, changes in physical activity levels, body weight or alcohol consumption are likely minimal. Together, these observations support that associations in this study could represent associations between lifestyle factors and breast cancer risk in PHTS patients. We found no indication for a different outcome of the effect of lifestyle factors on the breast cancer risk in PHTS patients compared to the general population or patients with other hereditary cancer predisposition syndromes. No indications to deviate from general recommendations were identified, and therefore, it is important for female PHTS patients to continue weekly physical activity, limit their alcohol consumption, limit smoking behavior and maintain their BMI in a healthy range. Maintaining a healthy lifestyle could potentially lower their breast cancer risk [6,8,14,21,26,31].

Although the observed effect sizes were similar to the general population, when observed associations represent causal relations of lifestyle to breast cancer risks, the absolute effects of lifestyle factors could potentially be higher due to the higher breast cancer risk in PHTS patients. Information on other factors that are known to influence the breast cancer risk, such as parity, breastfeeding, oral contraceptive use, menopause onset and weight changes, was not available [21]. Although this study already recruited PHTS patients from 12 countries and has the largest sample size to date, a follow-up study in a larger prospective cohort is recommended to assess causal associations and re-evaluate the reported small and moderate effects. Furthermore, it can subsequently be explored how PHTS could be included in the existing breast cancer risk prediction models to consolidate and guide personalized breast cancer risk management in PHTS patients [32].

## 5. Conclusions

Understanding cancer risk variation among PHTS patients is important to optimize patient counseling and cancer prevention programs. In this study, it is observed that the majority of female PHTS patients have an unhealthy BMI. Furthermore, it is suggested that breast cancer patients are about 1.04 to 1.3 times more often smokers than non-smokers and have 1.2 to 1.8 times more often a higher alcohol consumption. An overall healthy lifestyle by performing weekly physical activity, limiting alcohol consumption, limiting tobacco smoking and maintaining a healthy BMI could potentially reduce their breast cancer risk in a similar way as for patients in the general population. Together, these results suggest an important role for medical specialists to raise awareness about lifestyle among PHTS patients as a healthier lifestyle could potentially decrease their breast cancer risk. 

## Figures and Tables

**Table 1:** Characteristics of the study population. ^a^

	Including Breast Cancer Index	Excluding Breast Cancer Index
Characteristics	N = 125	N = 104
**Age at questionnaire completion, mean (SD)**	44 (13)	42 (13)
**Age at PHTS diagnosis, mean (SD)**	37 (14)	34 (14)
**Coding effect, N (%)**	124 (99%)	103 (99%)
Missense	51 (41%)	45 (44%)
Truncating	72 (58%)	57 (55%)
Other	1 (1%)	1 (1%)
**Domain, N (%)**	124 (99%)	103 (99%)
C2	32 (26%)	26 (25%)
Phosphatase	77 (62%)	64 (62%)
Other	15 (12%)	13 (13%)
**Breast cancer (invasive or in situ)**		
N (%)	39 (31%)	18 (17%)
Age, mean (SD)	43 (9)	44 (8)
**Preventive bilateral mastectomy ^b^**		
N (%)	16 (13%)	16 (15%)
Age, mean (SD)	33 (10)	33 (10)
**Lifestyle risk factors**		
**Physical activity, N (%)**		
<1 time a week	11 (9%)	8 (8%)
1 time a week	13 (10%)	10 (10%)
2–3 times a week	49 (39%)	43 (41%)
4–5 times a week	29 (23%)	25 (24%)
>5 times a week	23 (18%)	18 (17%)
**Tobacco smoking, N (%)**		
Currently smoking	6 (5%)	6 (6%)
Smoked in the past	22 (18%)	16 (15%)
Never smoked	89 (71%)	76 (73%)
Never smoked, but indoor smoking at home	8 (6%)	6 (6%)
**Smoking amount, N (%) ^c^**		
<10 per day	19 (68%)	15 (68%)
10-25 per day	9 (32%)	7 (32%)
>25 per day	0 (0%)	0 (0%)
**Weekday alcohol consumption, N (%)**		
0 glasses a day	115 (92%)	96 (92%)
1 glass a day ^d^	1 (1%)	1 (1%)
2 glasses a day	8 (6%)	6 (5%)
≥3 glasses a day	1 (1%)	1 (1%)
**Weekend alcohol consumption, N (%)**		
0 glasses a day	80 (64%)	65 (63%)
1 glass a day ^d^	0 (0%)	0 (0%)
2 glasses a day	35 (28%)	30 (29%)
≥3 glasses a day	10 (8%)	9 (9%)
**BMI (kg/m^2^), N (%)**		
<18.5 (underweight)	2 (2%)	2 (2%)
18.5–24.9 (normal weight)	32 (26%)	27 (26%)
25.0–29.9 (overweight)	38 (31%)	34 (33%)
≥30.0 (obesity)	50 (41%)	39 (38%)
Unknown	3	2

^a^ Characteristics are presented for the total study population (including breast cancer index) and for the cohort that is used for analyses corrected for ascertainment bias (excluding breast cancer index). ^b^ Preventive bilateral mastectomy includes patients with a bilateral mastectomy before breast cancer development or last follow-up. ^c^ For patients who smoke, or have smoked in the past, the number of cigarettes, cigars or tobacco pipes that they usually smoke(d) per day. ^d^ Including 0.5 glasses per day.

**Table 2:** Odds ratios for lifestyle factors and breast cancer. ^a^

	Including Breast Cancer Index Patients			Excluding Breast Cancer Index Patients		
Lifestyle Factor	n/N	OR (95%CI)	OR_adj_ (95%CI)	n/N	OR (95%CI)	OR_adj_ (95%CI)
**Physical activity**						
≥2 times per week	32/101	-	-	17/86	-	-
0–1 times per week	7/24	0.9 (0.3–2.3)	0.9 (0.3–2.6)	1/18	n/a	n/a
**Alcohol consumption**						
0–1 glass per day	32/108	-	-	14/90	-	-
≥1 glass per day	7/17	1.7 (0.6–4.7)	1.2 (0.4–4.0)	4/14	2.2 (0.5–7.6)	1.8 (0.4–6.9)
**Smoking**						
Non-smoking	27/97	-	-	12/82	-	-
Smoking	12/28	1.9 (0.8–4.6)	1.04 (0.4–2.8)	6/22	2.2 (0.7–6.6)	1.3 (0.3–4.2)
**BMI**						
Underweight or normal	11/34	-	-	6/29	-	-
Overweight or obese	27/88	0.9 (0.4–2.2)	0.98 (0.4–2.6)	12/73	0.8 (0.3–2.4)	0.8 (0.3–2.7)

^a^ Odds ratios (OR) with corresponding 95% confidence intervals (95%CI) per lifestyle factor are presented for females including breast cancer index patients (left) and for females excluding breast cancer index patients (right). ORs are presented without and with correction for the age at questionnaire completion (OR and OR_adj_, respectively). The upper group per lifestyle factor is the reference category. For each group, the number of patients with breast cancer (n) and the total number of patients with the lifestyle risk factor (N) are presented (n/N). Non-smoking includes patients who have never smoked and those who have never smoked but had indoor smoking at home. Smoking includes patients who reported to be current smokers or smokers in the past.

## Data Availability

Individual patient data cannot be shared due to privacy and ethical considerations. Requests for aggregate study data can be submitted to the corresponding author.

## Supplementary Materials

The following supporting information can be downloaded at: https://www.mdpi.com/article/10.3390/cancers16050953/s1, Table S1: Definition of coding effect. Table S2: Definition of PTEN protein domains.

## List of Abbreviations (Alphabetical Order)

BMI	Body mass index
OR	Odds ratio
PHTS	PTEN Hamartoma Tumor Syndrome
SD	Standard deviation
