# Supplementary material for: Lifestyle Factors and Breast Cancer in Females with PTEN Hamartoma Tumor Syndrome (PHTS)
Source: Cancers (Basel). 2024 Feb 27;16(5):953. doi: 10.3390/cancers16050953 (PMC10930780; doi:10.3390/cancers16050953)
Supplement: Supplementary file 1 [file cancers-16-00953-s001.zip › cancers-2839164-supplementary.pdf]

Supplementary Data

Supplement to:

Lifestyle Factors and Breast Cancer in Females with PTEN Hamartoma Tumor Syndrome (PHTS); Cancers; Linda A.J. Hendricks, Katja C.J. Verbeek, Janneke H. M. Schuurs-Hoeijmakers, Arjen R. Mensenkamp, Hilde Brems, Robin de Putter, Violetta C. Anastasiadou, Marie-Charlotte Villy, Arne Jahn, Verena Steinke-Lange, Margherita Baldassarri, Arvids Irmejs, Mirjam M. de Jong, Thera P. Links, Edward M. Leter, Daniëlle G.M. Bosch, Hildegunn Høberg-Vetti, Marianne Tveit Haavind, Kjersti Jørgensen, Lovise Mæhle, Ana Blatnik, Joan Brunet, Esther Darder, Emma Tham, Nicoline Hoogerbrugge, Janet R. Vos

Corresponding Author:

Janet R. Vos

Department of Human Genetics, Radboud university medical center, Nijmegen, The Netherlands

E-mail: janet.vos@radboudumc.nl

Supplementary Table S1. Definition of coding effect

Coding effect category	Definition
Truncating or predicted truncating	Frameshift, nonsense, start-loss, large structural variants (deletions of (the majority) of ≥ 1 exon), splice-site variants (+/- 5 base pairs (bp) of intron/exon boundary), and cryptic splice site variants
Missense	Missense and in-frame variants
Other	Variants in 5'- and 3'-UTR regions, synonymous variants, and intronic variants exceeding intron/exon boundary with more than 5bp

Supplementary Table S2. Definition of PTEN protein domains

Protein domain	Boundaries in amino acids (AA)
Phosphatase Binding Domain (PBD)	1-6
Phosphatase Domain (PD)	7-185
C2 domain (C2)	186-351
C-terminal Region (CTR)	352-400
PDZ domain (PDZ)	401-403
Other, including PBD, CTR, PDZ	Variants spanning multiple domains, not located in a domain, or located in domains other than PD or C2 were grouped.
